# Supplementary material for: Selective PPAR-Delta/PPAR-Gamma Activation Improves Cognition in a Model of Alzheimer’s Disease
Source: Cells. 2023 Apr 8;12(8):1116. doi: 10.3390/cells12081116 (PMC10136457; doi:10.3390/cells12081116)

Supplemental Figure S1: Markers for Neurotransmission

Markers for Neurotransmission

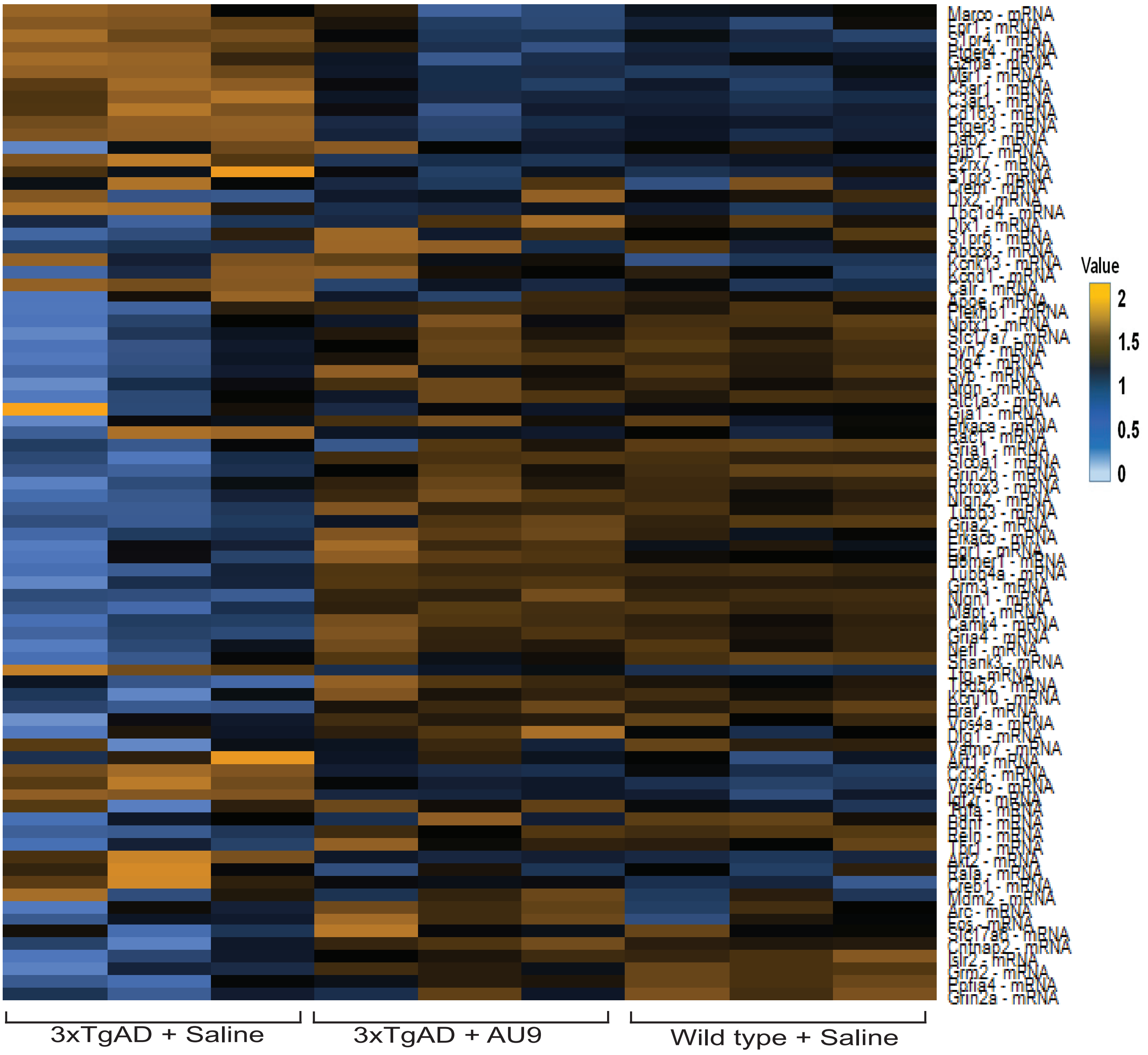

Supplemental Figure S2: Markers for cellular stress

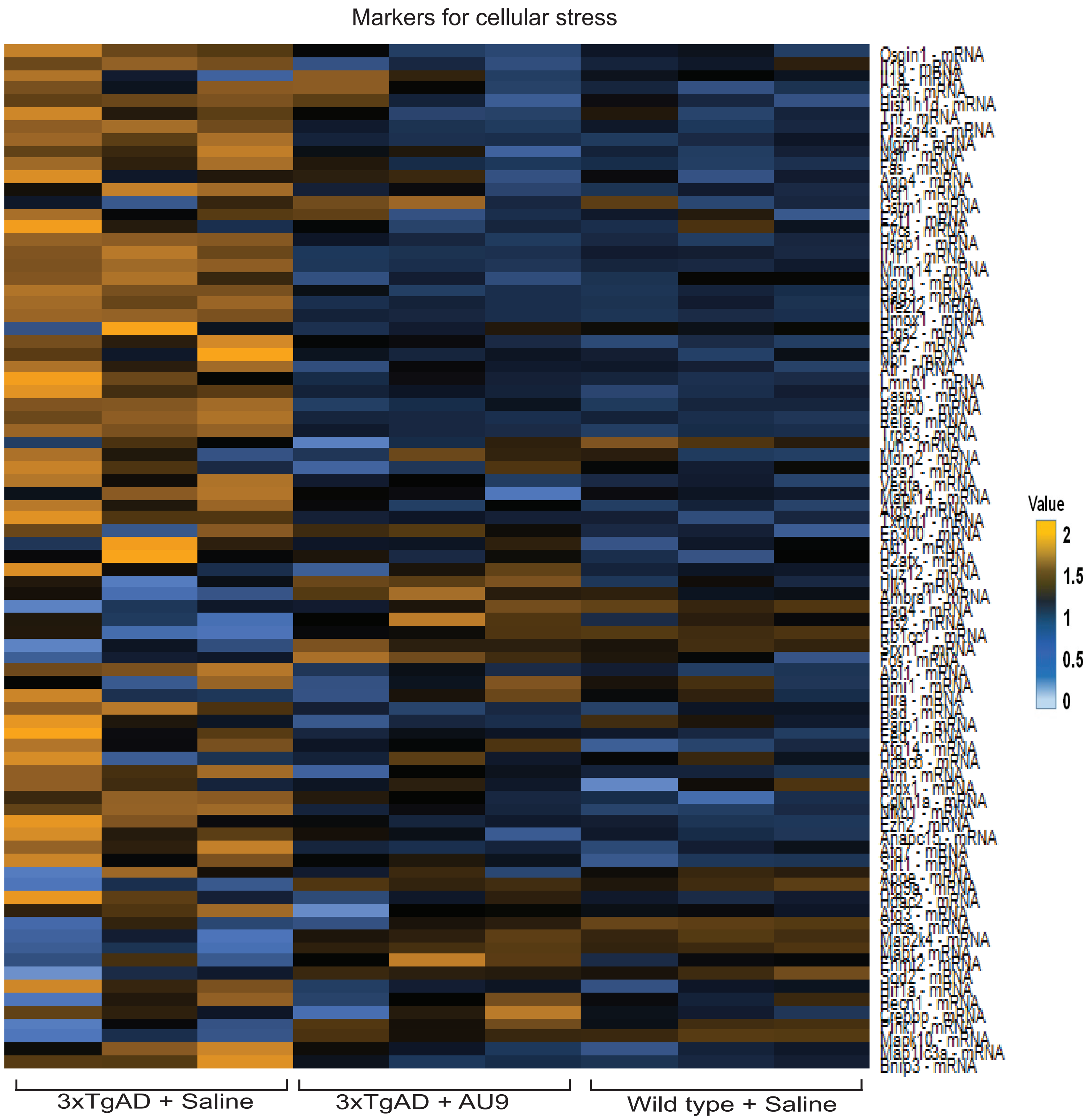

Supplemental Figure S3: Cytokines

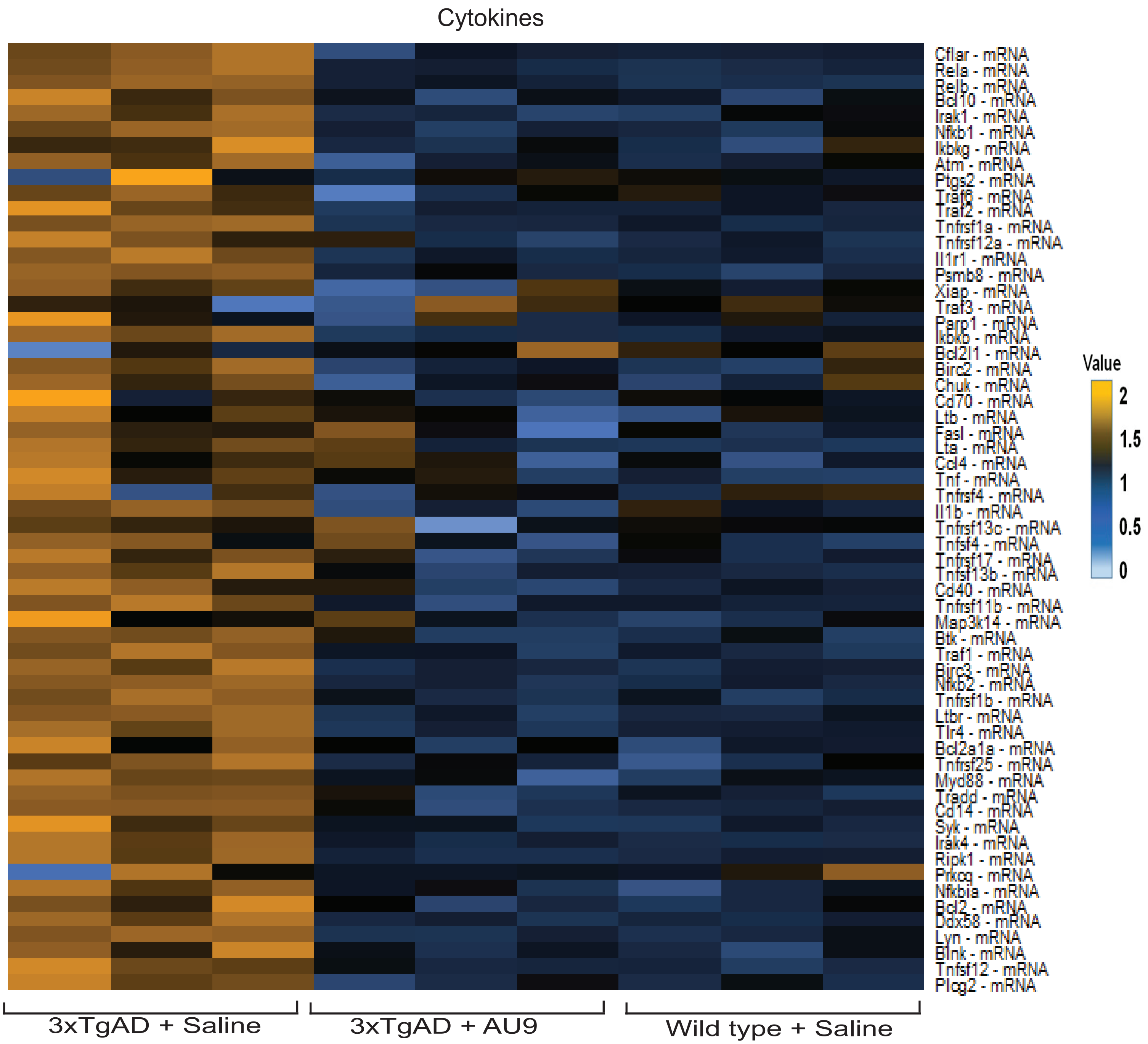

Supplemental Figure S4: Markers for DNA Damage

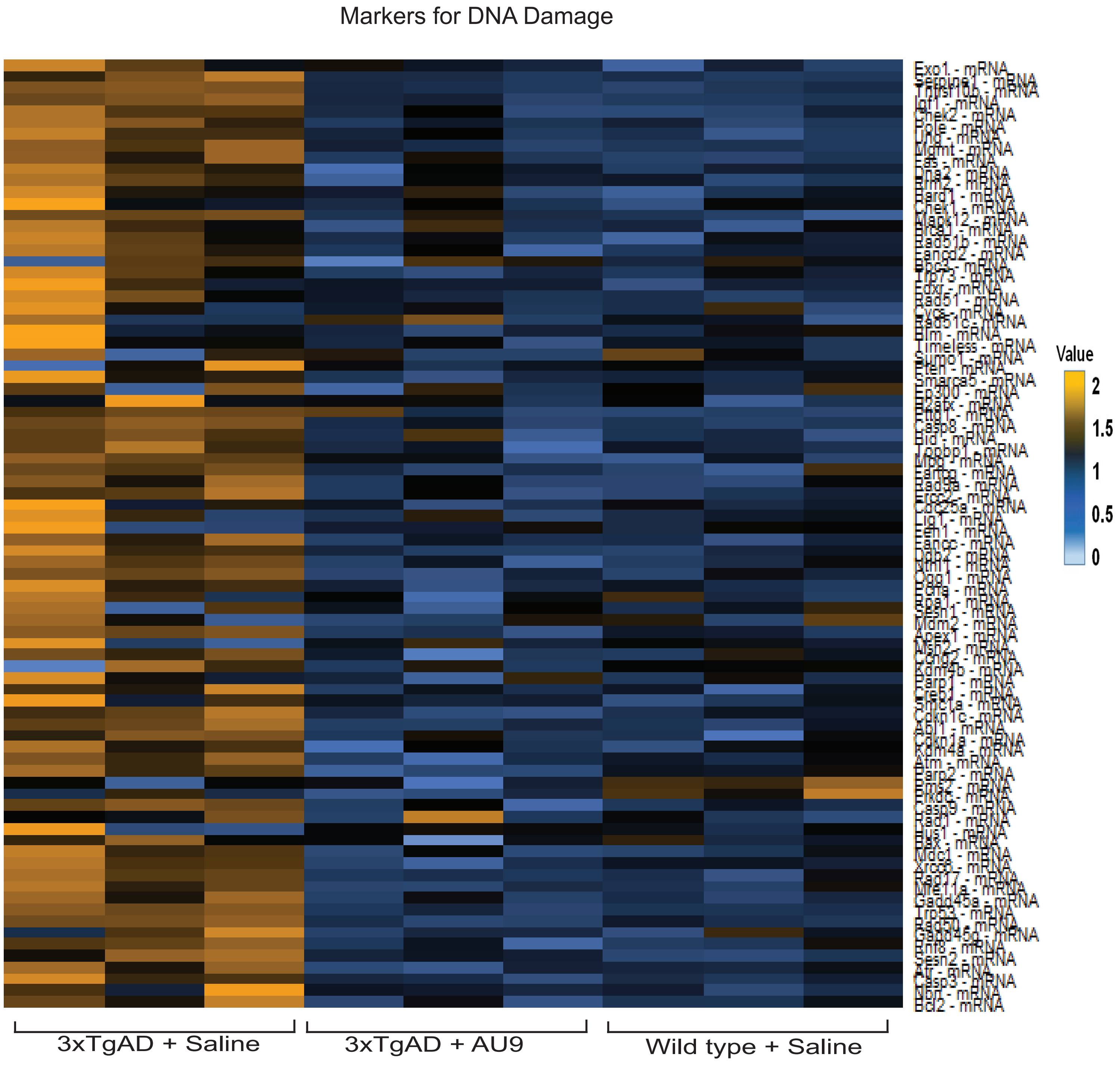

Supplemental Figure S5: QPCR primers for Figure 3C

QPCR primers for Figure 3C

**PDK1**	**GATAGTCTTGAGCCACCAAGTAG (Sense)**
	**CCACACAGGGACCACATAAA (AntiSense**
**CPT1b**	**TCTACCTCCCAAGTGCTAAGA (Sense)**
	**ACTCTCCTGGTCTGTGGATAA (AntiSense)**
**LCAD**	**GGTCAGGAGAGCTAATGGAAAG (Sense)**
	**GAGAGATGGCTCAGTGGTTAAG (AntiSense)**
**UCP1**	**GGTCAGGAGAGCTAATGGAAAG (Sense)**
	**GAGAGATGGCTCAGTGGTTAAG (AntiSense)**
**UCP3**	**GGTCAGGAGAGCTAATGGAAAG (Sense)**
	**GAGAGATGGCTCAGTGGTTAAG (AntiSense)**
**PGC1a**	**GGTCAGGAGAGCTAATGGAAAG (Sense)**
	**GAGAGATGGCTCAGTGGTTAAG (AntiSense)**
**FABP3**	**GGTCAGGAGAGCTAATGGAAAG (Sense)**
	**GAGAGATGGCTCAGTGGTTAAG (AntiSense)**
**LPL**	**CCCTGGAAGTGGGAAAGAATAG (Sense)**
	**GGTGCCTCTATCACAGCAATAA (AntiSense)**
**ACS**	**GTGTGTGTGTGTGTGTGTTATG (Sense)**
	**GAGAAGGGAGCCACAATTAAGA (AntiSense)**
**ILK**	**GAGCTTCTCCGAGGTTCATTAC (Sense)**
	**CAAAGCTGAAGGACAGAGGATAG (AntiSense)**
**UBC**	**GGGACTGCTGTGGTTTCATA (Sense)**
	**GGTGGACTCTTTCTGGATGTT (AntiSense)**
**PGAR**	**CAGAAAGAGGAGGCAGGTAAAG (Sense)**
	**CGTGGGATAGAGTGGAAGTATTG (AntiSense)**
**COX1**	**GAATAGTAGGCACCGCACTAAG (Sense)**
	**GGAGTAGCACGGCTGTAATAAG (AntiSense)**
**NRF1**	**CCCAGAACAAGACCCAGTAAG (Sense)**
	**TCCTGAGGGAGTACCAGAATAG (AntiSense)**
**TFAM**	**CGGCCACTGTGTACTCATATAC (Sense)**
	**CCATCTCCATTTCTCCCTCATT (AntiSense)**

Supplemental Figure S6: QPCR primers for Figure 8A (1)

QPCR primers for Figure 8A

**ADCy8**	**GCGGGATGGGATGGAATAAA (Sense)**
	**GTGAGGCAAGCCAGTAAAGA (AntiSense)**
**Adra2a**	**CTCGCTGAACCCTGTTATCTAC (Sense)**
	**GACCGCCCTGAATGATCTTTAT (AntiSense)**
**C1qa**	**GGAGAGTAGGCAGATGTTTGT (Sense)**
	**CCAGTCTAGGGCCATTCATATC (AntiSense)**
**C1qb**	**CCAGAGCACCAATCCATCTT (Sense)**
	**GCCTACCCACTTCTTCATCTATC (AntiSense)**
**CD33**	**CCCACCTAGCCAAGATTCTAAC (Sense)**
	**GGCGTCTCTTCACAGCAATA (AntiSense)**
**Csf1r**	**CCCTCTAACCAGGGTCTCTAA (Sense**
	**CACCAGGGTGAGTCTTCTAATG (AntiSense)**
**Csf2rb**	**GGGCTCTGTCTGGTCAATATAAG (Sense)**
	**GCACGAGTATTCAGTCCCTAAC (AntiSense)**
**Ctse**	**AGGAACCACCTAGTCCTCTATC (Sense)**
	**AGTAGGCGCATTGATCCTTATC (AntiSense)**
**Trem2**	**GTGGTAGTGCCAGTAGTGTAAAG (Sense)**
	**GGGCTCCAGTCTTGTGTAAAT (AntiSense)**
**IGF1r**	**CCAAAGACGAGAGAGGTAGAAAG (Sense)**
	**CAGAGGGCTGTTAGGATGTATG (AntiSense)**
**Cxxl11**	**GCATCCAGGTAGTGACCATAAA (Sense)**
	**CCTAGAGGAGGTGTAGAGAGAAA (AntiSense)**
**Cscl16**	**CACCTGAAACCGGGACATAATA (Sense)**
	**TCTCTCTCTCTCTCTCTCTCTCT (AntiSense)**
**Cybb**	**CACCCATTGTCTCCTTCTACTC (Sense)**
	**CTGTCCTTCTCTCTTCCTCATTC (AntiSense)**
**Il13ra1**	**GCACTGGATGCTGTCTTATCT (Sense)**
	**CATGCCTATCTCTGAAGGTTGT (AntiSense)**
**Il1b**	**TCAGGAATGGAGGGCTATCT (Sense)**
	**AGTGTGTGGGTTGCCTTATC (AntiSense)**

Supplemental Figure S7: QPCR primers for Figure 8A (2)

QPCR primers for Figure 8A

**Il1r1**	**CTCTGTACCTGCTCTTGGAATG (Sense)**
	**GCCTGGGTCCAGTAGAATTAAG (AntiSense)**
**Il10ra**	**GGCTTTCTGGATGTGGTAAGA (Sense)**
	**CTGGGTCTGCAGTTAGAGATTAG (AntiSense)**
**HGF**	**GTTGTCAGGTTCCCAGGAATAG (Sense)**
	**GGAAGGATGGAAGGAGCTAAAG (AntiSense)**
**Inhbb**	**CAGCAGAGTCCATTCCCTATTG (Sense)**
	**CCTCACAGTAGTTCCCGTAGTA (AntiSense)**
**Itga5**	**GAAGAGGAAGGGCTAGGATAGA (Sense)**
	**GGAGACACTGAAGAGGAGGATA (AntiSense)**
**Lif**	**TGGCTTCATCACTCCACTATTC (Sense)**
	**CAGGTGCTTCTTACAGGTCTAC (AntiSense)**
**MMP24**	**AAGGACTGTGTCTTCTGTCTTG (Sense)**
	**CAGGTTGGAGGGAGATGTTTAG (AntiSense)**
**MMP9**	**CCAATCTTAGCCTCTGCCTATC (Sense)**
	**GATGCCGTCTATGTCGTCTTTA (AntiSense)**
**NGFr**	**CGCTCTTGATGGACACTTATCT (Sense)**
	**GCTGTTGGCTCCTTGTTTATTT (AntiSense)**
**P2ry12**	**GGTCAGAAGTGAGGCAGTAAG (Sense)**
	**TTCCCTGTGGTTGGGTAAAG (AntiSense)**
**Scamp2**	**CTGGAGAGGTGTCTTGCTATTC (Sense)**
	**GGCTGGCCCAGAACTTATT (AntiSense)**
**Sla**	**CTTCCCTGGATGGTGTGTATTT (Sense)**
	**GGTGACAAGTGAGTGGGAATAG (AntiSense)**
**TNF**	**AGGGAAAGAAGTGGGCTAATG (Sense)**
	**CTGCCCTTCCTCCATCTTAAA (AntiSense)**

Supplemental Figure S8: Western Whole blots for PSD95, GluA1, GluA2, and the associated standardizing Al-pha-Actin

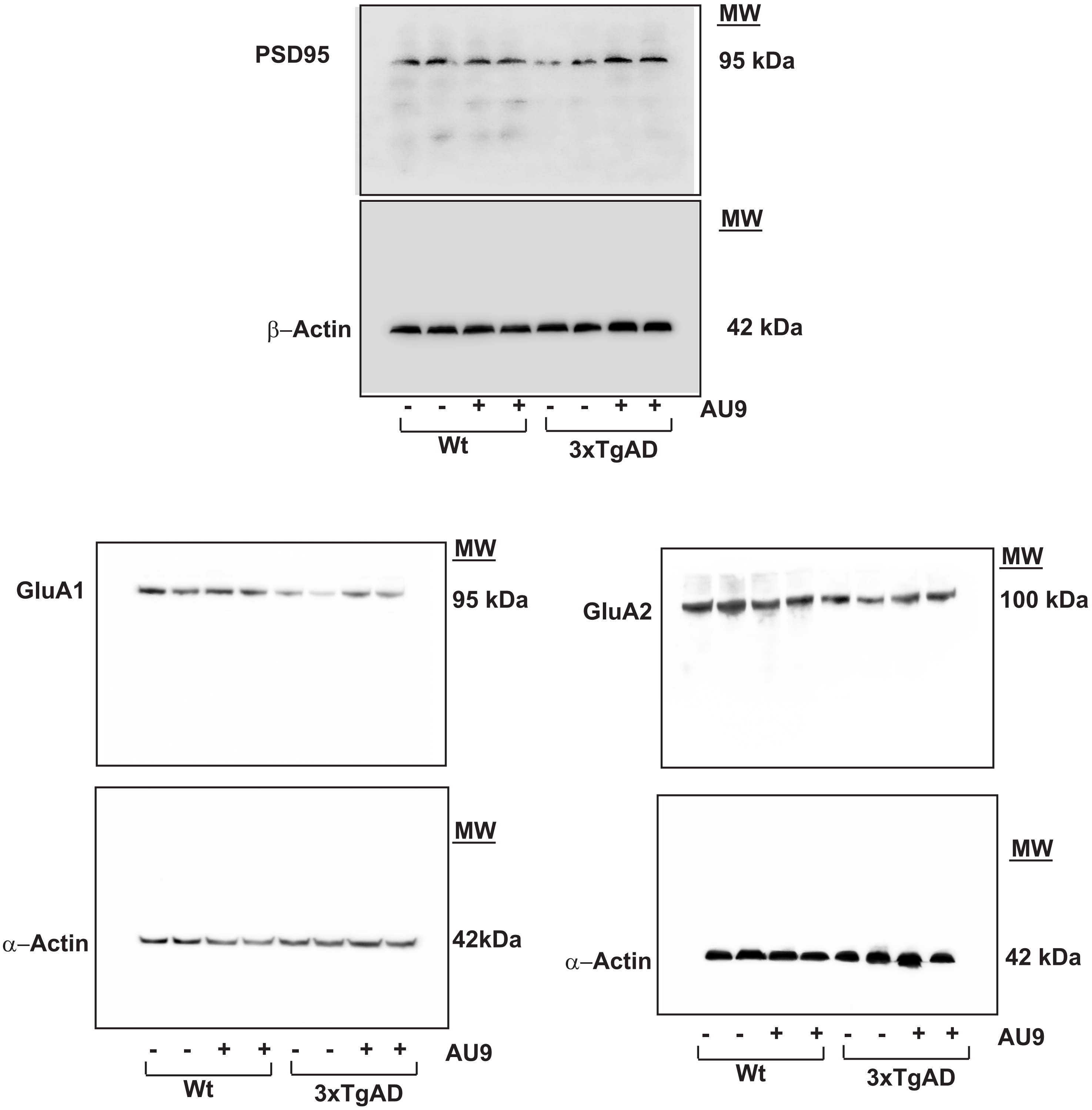

Supplemental Figure S9: Western whole blots for inflammatory markers

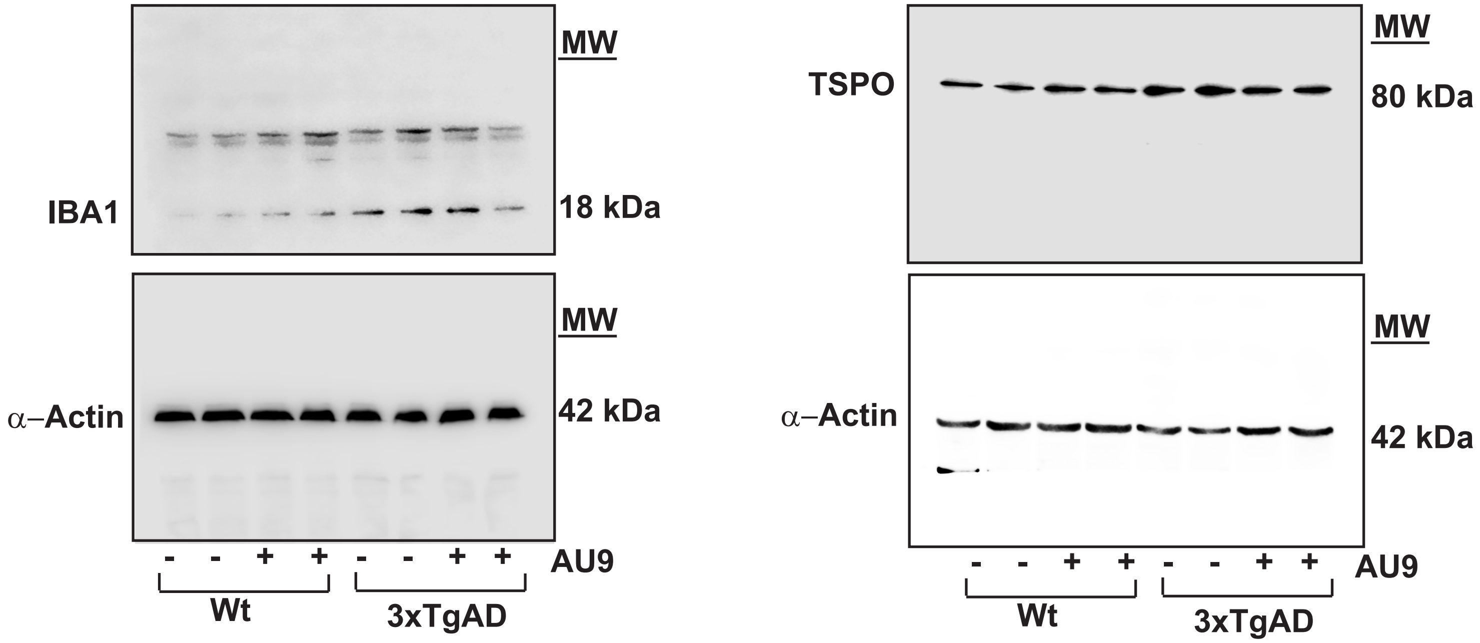

Supplement: Supplementary file 1 [file cells-12-01116-s001.zip › cells-2168499-supplementary.pdf]
